# The treatment of migraines and tension-type headaches with intravenous and oral niacin (nicotinic acid): systematic review of the literature

**DOI:** 10.1186/1475-2891-4-3

**Published:** 2005-01-26

**Authors:** Jonathan Prousky, Dugald Seely

**Affiliations:** 1 Department of Clinical Education, The Canadian College of Naturopathic Medicine, 1255 Sheppard Avenue East, Toronto, Ontario, M2K 1E2, Canada; 2 Department of Research, The Canadian College of Naturopathic Medicine, 1255 Sheppard Avenue East, Toronto, Ontario, M2K 1E2, Canada; 3 Institute of Medical Science, University of Toronto, Toronto, Canada

## Abstract

**Background:**

Migraine and tension-type headaches impose a tremendous economic drain upon the healthcare system. Intravenous and oral niacin has been employed in the treatment of acute and chronic migraine and tension-type headaches, but its use has not become part of contemporary medicine, nor have there been randomized controlled trials further assessing this novel treatment. We aimed to systematically review the evidence of using intravenous and/or oral niacin as a treatment for migraine headaches, tension-type headaches, and for headaches of other etiologic types.

**Methods:**

We searched English and non-English language articles in the following databases: MEDLINE (1966–February 2004), AMED (1995–February 2004) and Alt HealthWatch (1990–February 2004).

**Results:**

Nine articles were found to meet the inclusion criteria and were included in this systematic review. Hypothetical reasons for niacin's effectiveness include its vasodilatory properties, and its ability to improve mitochondrial energy metabolism. Important side effects of niacin include flushing, nausea and fainting.

**Conclusion:**

Although niacin's mechanisms of action have not been substantiated from controlled clinical trials, this agent may have beneficial effects upon migraine and tension-type headaches. Adequately designed randomized trials are required to determine its clinical implications.

## Introduction

Migraines and tension-type headaches impose an important burden upon society and the working public. According to the National Headache Foundation, some 45 million Americans suffer from chronic, recurring headaches and 28 million of these suffer from migraine headaches annually [1]. Furthermore, the work force loses approximately 50 billion dollars per year due to absenteeism and medical expenses caused by headaches, with more than 157 million workdays lost each year to migraine sufferers alone [1].

Even though advances have been made with regard to the treatment of acute migraine headaches (i.e., the triptan formulations), many patients often discontinue their migraine interventions due to treatment dissatisfaction [2]. Among individuals seeking treatment for tension-type headaches, the frequency of such headaches is often daily or almost every day [3]. Unfortunately, chronic tension-type headaches are associated with analgesic abuse [4], and are difficult to manage in a primary care setting due to frequent comorbid psychiatric or analgesic use problems [5]. Thus, it is imperative that other methods of treatment be researched and developed in order to increase satisfaction, therapeutic response, and compliance amongst these patients.

One novel, but not really new treatment option, is the administration of niacin (nicotinic acid) through intravenous and/or oral routes. Niacin is a well-known over-the-counter (OTC) supplement primarily used for its ability to favorably influence cholesterol levels. Recently, there have been anecdotal reports demonstrating the effectiveness of niacin for aborting acute migraine attacks [6], and for preventing migraine headaches [7]. To assess the therapeutic spectrum of niacin's clinical effectiveness, we conducted a systematic review of the literature to examine the evidence of intravenous and/or oral niacin as a treatment for migraine headaches, tension-type headaches, and for headaches of other etiologic types.

## Methods

### Literature Search

We conducted a systematic search of English and non-English language articles in the following databases: MEDLINE (1966–February 2004), AMED (1995–February 2004) and Alt HealthWatch (1990–February 2004). Articles were searched with the key search terms "Migraine" combined with the Boolean Operator "AND "Niacin" OR "Nicotinic Acid." Additional searches were conducted with the search terms "Headache" and "Tension." To supplement the search, we searched through the references of the articles we found from the databases.

### Selection of Articles

To be included in our final review, articles had to report on the use and administration of niacin for migraine or any other types of headache. We included articles assessing original reports in both peer-reviewed and non-peer-reviewed journals.

### Quality assessment

We determined the evidence grade of each report found, based on the hierarchy of evidence developed by the Oxford Centre for Evidence Based Medicine [8]. Table 1 displays the hierarchy of evidence.

**Table 1:** Grades of Evidence

**A**	Systematic reviews of randomized controlled trials and/or randomized controlled trials.
**B**	Systematic reviews of observational studies and/or high-quality observational studies including cohort and case-control studies.
**C**	Case-series, case-reports and/or poor quality cohort and case-control studies.
**D**	Expert opinion without explicit critical appraisal, or based on physiology, bench research or "first principles."

### Search Results

A total of 14 articles were screened [6,7,9-20]. Five articles were excluded in total; three because niacin was not the sole therapeutic agent used for the treatment of headache [16], histaminic cephalgia [17], and migraine [20]; and two because the reports were opinion pieces without any objective or subjective data to support the assertions made [13,19]. In total, nine articles were found to meet the inclusion criteria and were included in this systematic review [6,7,9-12,14,15,18]. Table 2 displays the characteristics of the studies included in this review.

**Table 2:** Summary of Articles Demonstrating Niacin's Effectiveness for the Treatment of Migraine Headaches, Tension-type Headaches, and Headaches of other Etiologies

**Reference**	**Condition**	**n**	**Protocol**	**Outcome**	**Evidence Grade**
9	Migraine headaches	21	One initial intramuscular injection (IM) followed by a series of 6 or 8 intravenous (IV) treatments (maximum 50 mg), then regular IM injections (25–50 mg) combined with 50–150 mg of oral administration.	17 of the 21 subjects had a positive response.	C: case-series
10	Headaches of different etiologic types	100	100 mg of IV sodium nicotinate or niacin.	75 of the 100 subjects had complete relief.	C: case-series
11	Migraine headaches	15	100 mg of IV niacin, and an additional 50–200 mg if necessary to ensure a flushing response of more than 15-minutes.	13 of the 15 subjects had a positive response. The headaches were relieved in 27 of the 31 times when niacin was administered by IV administration.	C: case-series
12	Tension headaches	35	22 subjects received 100–200 mg of IV niacin for a total of 53 times.	13 of the 22 subjects had a positive response. The headaches were relieved in 41 of the 53 times when niacin was administered by IV administration.	C: case-series
14	Emotional or tension headaches	5	100 mg of IV niacin regularly for 12 weeks combined with a graded schedule of oral dosing, beginning at 300 mg daily, increasing to 900 mg daily, and tapering down to 300 mg daily.	All 5 cases of emotional or tension headaches were very responsive to both IV and oral niacin.	C: case-series
15	Tension headaches accompanied with depression	50	100 mg of IV niacin regularly for 23 weeks and then continued once every 2 months and as needed. This was combined with a graded schedule of oral dosing, beginning at 300 mg daily, increasing to 900 mg daily, and tapering down to 300 mg daily.	In 44 of the 50 subjects the results with niacin therapy were very satisfactory or favorable.	C: case-series
18	Migraine headaches	1	300–500 mg of niacin were chewed and allowed to slightly dissolve in the mouth.	Resolution of migraine headaches.	C: case-report
6	Migraine Headaches	2	500 mg of oral niacin taken at the onset of acute symptoms.	In 2 of the 2 subjects, niacin aborted the acute migraine symptoms. In the first subject, niacin resolved the acute attacks in 4 of 4 occasions. In the other subject, niacin resolved the attack on one occasion.	C: case-report
7	Migraine headaches	1	375 mg of oral sustained-release niacin twice daily for 1 month, and 375 mg once daily for 2 months.	Migraine-free for the first month, and a marked reduction in migraine headaches over the next 2 months.	C: Case-report

## Limitations

### Article #1 [9]

This case series of 21 patients was limited as it was uncontrolled and involved a small number of patients. The methods that were used to evaluate efficacy of the treatment were primarily based upon a subjective questionnaire or medical record. The results would have been more meaningful if all the patients used the questionnaire to evaluate their treatment responses. Finally, although the symptomatic changes were witnessed immediately following niacin injection, the lifestyle recommendations may have had therapeutic benefits as well.

### Article #2 [10]

This study involved 100 patients having headaches of multiple etiologies. This study was not properly controlled, but did at least provide some comparison against a group of patients that were not administered the flush forms of intravenous niacin. The fact that the control group did not substantially benefit from the intravenous niacinamide lends more therapeutic efficacy to the ability of intravenous sodium nicotinate or niacin to have a marked therapeutic effect. The sample size was sufficient in number (n = 100), but the determination of therapeutic effectiveness was purely subjective and did not include any questionnaire or standardized method of evaluation.

Side effects were minimal; 2 patients experienced mild abdominal cramps, 1 patient vomited but was suspected as having a pathologic condition of the stomach, and 1 other patient with migraine vomited forty-five minutes after the injection. A few patients found the treatment to be worse than their headaches. The authors concluded that the relief of headaches seemed to be correlated with the degree of flushing from the sodium nicotinate or niacin, and that this therapy was most useful among the migraine, spinal tap, and idiopathic groups.

### Article #3 [11]

A number of shortcomings were evident in this article. The first of which was that the study was not properly controlled and did not contain a placebo group or a group of patients acting as self-controls. However, some of the patients were given intravenous treatments on more than one occasion. This procedure helped in determining treatment reliability and reproducibility since niacin appeared to achieve therapeutic benefits on several occasions. There were no standardized methods of evaluating efficacy since the treatment responses were based upon the medical record and subjective reports. The results would have been more meaningful if all the patients used a standardized questionnaire prior to and after each treatment. In addition, there was only one male subject and 14 females in the study.

### Article #4 [12]

In this study of 35 patients were given treatments of intravenous dihydroergotamine methanesulfonate, intravenous niacin, or oral combination tablets of ergotamine tartrate and caffeine. Although no control group was used in this study, the patients were given multiple treatments on several occasions. This offered an interesting comparison to be made between niacin and other treatments. Since no control or placebo group was included, it cannot be determined if the therapeutic results were due, in part, to chance or placebo effects. The results might have been more meaningful if all the patients were given a standardized questionnaire prior to and after each treatment, and if an objective measure was incorporated to further substantiate patient responses.

### Article #5 [14]

These cases reported were well described and clearly demonstrated therapeutic responses during the intravenous and oral niacin therapy. The results would have been more reliable if a comparison had been made to a control group or to a similar patient cohort that were not given the same treatments. Even if the patients served as self-controls, and were told to stop the niacin treatment for a specified period of time, more information could have been gained from their therapeutic responses to niacin. Overall, this report of five cases provides an interesting approach to patients having chronic tension headaches and depression. Its value is limited by the difficulty in extrapolating these findings to a greater number of patients.

### Article #6 [15]

In this study involving 50 patients there was no data that listed pertinent identifying information, treatment response, past treatments, and duration of treatment for each patient. This would have strengthened the report by being more descriptive, and thus more amenable to critical analysis. If a comparison were made to a control group or a similar patient cohort not given the same treatments, more validity could be have been ascribed to this method of treatment. The patients could have also served as their own controls, thus providing more information about the therapeutic responses to niacin. It cannot be determined if the therapeutic results were due, in part, to chance or placebo. No method of evaluating efficacy was mentioned, except that the responses to niacin were "very satisfactory" in 44 of 50 cases. The results would have been more meaningful if all the patients were given a standardized questionnaire prior to and after each treatment, and if an objective measure was incorporated to substantiate their responses to niacin.

### Article #7 [18]

In this report, Hall describes the use of niacin for his migraine headaches remarking that the migraines resolved when intense flushing occurred. According to Hall, niacin's benefits and side effects might be due to its ability to release serotonin and histamine from the stomach. There is no reason to doubt Hall when describing his therapeutic response to niacin. However, his report was brief, had no control and was entirely subjective as he was the participant as well as examiner.

### Article #8 [6]

In this report of 2 cases, it was found that in both cases migraines were relieved with oral niacin. The report would have been more rigorous if the patients had acted as their own self-controls. The value of the report is further diminished by the difficulty in extrapolating these findings to a greater number of patients.

### Article #9 [7]

This case report was of a 62-year-old woman with a 40-year history of migraine headaches. The patient never acted as her own self-control, which would have made the findings of the case report more meaningful. The fact that the patient's migraine headaches increased in severity after a reduction in dosage does lend more support to niacin as being a migraine preventive agent. The hypothesized increase in serotonin from niacin administration cannot be proven given the limited amount of data contained in the case report. Like all case reports, its value is diminished by the difficulty in extrapolating these findings to a greater number of patients.

## Discussion

The results of this systematic review indicate that niacin may have a therapeutic effect on migraine headaches, tension-type headaches, and headaches of other etiologies. The quality of the evidence at this point, however, is only hypothesis generating, and randomized trials are required to determine the clinical implications of this novel treatment.

There are several important limitations to consider in the interpretation of this review. We did not find any randomized or controlled trials of niacin on these headaches. We cannot determine to what extent publication bias has on the results of this review. We are unable to draw clinical inferences on the results of the included studies as they were of low quality and have a low level of external generalizability. Despite these limitations, we attempted to conduct an exhaustive search and included all reports of relevance.

Reasons for niacin's effectiveness can only be considered hypothetical, and require clarification from future randomized controlled trials. In acute migraine headaches some of the symptoms arise from activation of the trigeminovascular complex. Activation of this complex leads to intracranial vasoconstriction causing the migraine aura, followed by headache due to vasodilation of the extracranial vessels and activation of the perivascular nociceptive nerves [21]. When taken intravenously or orally, niacin causes cutaneous flushing that might abort the acute symptoms of migraine by vasodilating the intracranial vessels, thus preventing the subsequent vasoconstriction of the extracranial vessels. There is evidence that niacin is an effective peripheral vasodilator, but its ability to influence central mechanisms (i.e., cerebral blood flow and cranial hemodynamics) involved in migraine headaches have not been well studied. Niacin causes peripheral vasodilation and cutaneous flushing by inducing the production of prostaglandin D_2 _(PGD_2_) in the skin, leading to a marked increase of its metabolite, 9α, 11β-PGF_2_, in the plasma [22]. When niacin is administered orally in amounts of 500 mg or topically via a 6-inch patch of 10^-1 ^M aqueous methylnicotinate on the forearm, PGD_2 _is markedly released in the skin and its metabolite appears in high amounts in the plasma [22,23]. It is not known if PGD_2 _causes vasodilation of the intracranial arteries, but niacin's ability to abort acute migraine headaches suggests that this might be what is occurring. Old reports cited by Bicknell and Prescott [24], demonstrate that niacin does indeed cause vasodilation of the cerebral and spinal vessels, and that intravenous administration increases the rate of intracranial blood flow in human beings for 20–60 minutes without any significant change in blood pressure. Unfortunately, there have not been more recent reports examining the effects that niacin has upon cerebral blood flow in human subjects.

In terms of tension-type headaches, it appears that intravenous niacin is of benefit acutely due to its presumed central vasodilatory properties. Like migraines, part of the underlying pathophysiology of chronic tension-type headaches involves central mechanisms, such as the trigeminal system [26]. Chronic tension-type headaches are also associated with cerebrospinal pressure or intracranial venous pressure (or both) [26]. In fact, tension-type headaches are more similar to migraine headaches than they are dissimilar, in that they seem to progress into migraine headaches due to an escalating pathophysiological process [27]. Thus, niacin might mitigate the acute phase of tension-type headaches through the same hypothesized mechanism of action described earlier.

Some of the reports did demonstrate prophylactic benefits when niacin was administered orally every day. It is now recognized that a deficit of mitochondrial energy metabolism (i.e., impaired mitochondrial phosphorylation potential) plays a role in the pathogenesis of chronic migraine headaches [28]. Niacin maintains adequate mitochondrial energy metabolism by increasing substrate availability to complex I [29], and this is how it might function as an effective prophylactic agent for migraine prevention. Two other nutritional agents (riboflavin and coenzyme Q10) augment complex I of the mitochondrial respiratory chain, and have been subjected to clinical trials demonstrating their effectiveness for the prevention of migraine headaches [30-32]. A deficit of mitochondrial energy metabolism may play a role in the pathogenesis of migraine. Since niacin improves mitochondrial energy metabolism by increasing substrate availability to complex I, it might also be an effective agent for migraine prevention.

Niacin might also prevent tension-type headaches by improving mitochondrial energy metabolism within the skeletal muscles, and by increasing blood flow and oxygenation to the skeletal muscles. The overall net-effect could be a reduction in lactic acid concentrations, leading to reduced episodes of muscular tension and soreness. Niacin may reduce lactic acid concentrations since supplemental niacinamide (the amide of niacin) has been shown to reduce blood lactate and pyruvate concentrations by more than 50% in a patient with MELAS (mitochondrial encephalopathy, myopathy, lactic acidosis, and stroke-like episodes) syndrome by the third day of treatment [33]. This possible mechanism might only relate to migraine sufferers, however, since plasma levels of lactic and pyruvic acids were found to be significantly higher in migraine patients compared to patients with tension-type headaches and normal controls [34].

The side effects of intravenous niacin were found to be minimal from the summarized articles. The most common side effects were abdominal cramping, vomiting, and uncomfortable sensations of the skin and burning. A few of the patients described found the treatments to be worse than their headaches. In the articles where blood pressures were evaluated, little-to-no change occurred among the individuals treated with intravenous niacin. In a more recent study, parenteral niacin given to hypertensive and normotensive patients demonstrated a significant decrease in systolic, diastolic, and pulse pressures among the hypertensive subjects [35].

With respect to the oral administration of niacin, very few patients reported side effects. Even though niacin was well tolerated orally, we previously reported in a randomized placebo-controlled trial examining the safety of immediate-release or crystalline niacin, that it can be associated with intolerable side effects [36]. The most common side effects found using 500 mg of immediate-release niacin were unpleasant warmth or flushing, pruritis, chills, tingling, nausea, and vomiting. Approximately 75% of the subjects who were given niacin found it tolerable or difficult to tolerate, but did not indicate that they would never take niacin again. Some 18.2% of the niacin subjects indicated that they found niacin to be intolerable and would never take it again [36]. By contrast, very few of the patients from the summarized articles discontinued treatment due to the side effects of oral niacin.

The side effects of greater concern from oral niacin have to do with sustained- or slow-release preparations. These preparations are better in terms of compliance since patients experience less cutaneous flushing with them. However, the use of these preparations have been associated with reversible hepatic toxicity in doses equal to or greater than 1500 mg per day with an acute onset of clinical symptoms of hepatitis in a relatively short period of time (2 days to 7 weeks) [37]. Other reports have demonstrated clinical symptoms of hepatitis when much larger doses of sustained-release niacin (greater than or equal to 3 grams per day) were used for months to years [38-42]. Sustained-release preparations have a higher incidence of hepatic toxicity in doses comparable to the immediate-release preparations [43], but these important differences are not typically mentioned in reviews of niacin's lipid lowering properties.

## Conclusion

Even though niacin's mechanisms of action have not been substantiated from controlled clinical trials. It is possible that this agent has a beneficial effect upon migraine and tension-type headaches and the prophylaxis of these headaches. It is imperative that properly designed controlled trials are developed in order to determine niacin's true therapeutic effects and adverse effect profile. In terms of its ability to abort acute migraine headaches, a placebo-controlled trial of parenteral niacin and sumatriptan seems to be worthy of consideration. In terms of prophylaxis, a placebo-controlled trial of oral niacin and riboflavin or coenzyme Q10 also seems worthy of investigation.

## Competing interests

Dr. Jonathan Prousky is associated with Swiss Herbal Remedies, Ltd., a company that sells nutritional supplements including niacin. They had knowledge of this manuscript and have not seen this manuscript.

## Authors' contributions

JP wrote the manuscript. DS contributed to the text, revised the results section, and assisted with the tables.

## References

[B1] National Headache Foundation. http://www.headaches.org/consumer/generalinfo/factsheet.html.

[B2] Dodick DW (2003). Patient-focused migraine management: establishing needs. Drugs Today (Barc).

[B3] Holroyd K, Stensland M, Lipchik G, Hill K, O'Donnell F, Cordingly G (2000). Psychosocial correlates and impact of chronic tension-type headaches. Headache.

[B4] Granella F, Farina S, Malferrari G, Manzoni GC (1987). Drug abuse in chronic headache: a clinico-epidemiologic study. Cephalalgia.

[B5] Schoenan J, Wang W, Goadsby PJ, Silberstein SD (1997). Tension-type headache. Headache.

[B6] Prousky J, Sykes E (2003). Two case reports on the treatment of acute migraine with niacin. Its hypothetical mechanism of action upon calcitonin-gene related peptide and platelets. J Orthomol Med.

[B7] Velling DA, Dodick DW, Muir JJ (2003). Sustained-release niacin for prevention of migraine headache. Mayo Clin Proc.

[B8] Centre for Evidenced-Based Medicine. http://www.cebm.net/levels_of_evidence.asp#levels.

[B9] Atkinson M (1944). Migraine headache: some clinical observations on the vascular mechanism and its control. Ann Intern Med.

[B10] Goldzieher JW, Popkin GL (1946). Treatment of headaches with intravenous sodium nicotinate. JAMA.

[B11] Grenfell RF (1949). Treatment of migraine with nicotinic acid. Am Pract.

[B12] Grenfell RF (1951). Treatment of tension headache. Am Pract Dig Treat.

[B13] Cutter EP (1953). The management of headache. J Tn State Med Assoc.

[B14] Morgan ZR (1953). Nicotinic acid therapy in vasoconstriction type of headache. Md State Med J.

[B15] Morgan ZR (1955). A newer method of nicotinic acid therapy in headache of the vasoconstrictive type. J Am Geriatr Soc.

[B16] Ogden HD, Ching CL (1962). Clinical study of headache relief with a niacin-containing compound. Headache.

[B17] Ryan RE (1963). Modern concepts of the management of histaminic cephalgia. South Med J.

[B18] Hall JA (1991). Enhancing niacin's effect for migraine. Cortlandt Forum.

[B19] Hendler SS (1991). The Doctor's Vitamin and Mineral Encyclopedia.

[B20] Gedye A (2001). Hypothesized treatment for migraine using low doses of tryptophan, niacin, calcium, caffeine, and acetylsalicylic acid. Med Hypotheses.

[B21] eMedicine. http://www.emedicine.com/neuro/topic517.htm.

[B22] Morrow JD, Parsons WG, Roberts LJ (1989). Release of markedly increased quantities of prostaglandin D2 in vivo in humans following the administration of nicotinic acid. Prostaglandins.

[B23] Morrow JD, Awad JA, Oates JA, Roberts LJ (1992). Identification of skin as a major site of prostaglandin D_2 _release following oral administration of niacin in humans. J Invest Dermatol.

[B24] Bicknell F, Prescott F (1953). The Vitamins In Medicine.

[B25] Nardone R, Tezzon F (2003). The tirgemino-cervical reflex in tension-type headache. Eur J Neurol.

[B26] Hannerz J, Jogestrand T (2004). Relationship between chronic tension-type headache, cranial hemodynamics, and cerebrospinal pressure: study involving provocation with sumatriptan. Headache.

[B27] Cady R, Schreiber C, Farmer K, Sheftell F (2002). Primary headaches: a convergence hypothesis. Headache.

[B28] Tepper SJ, Rapoport A, Sheftell F (2001). The pathophysiology of migraine. Neurologist.

[B29] Marriage B, Clandinin MT, Glerum DM (2003). Nutritional cofactor treatment in mitochondrial disorders. J Am Diet Assoc.

[B30] Schoenen J, Lenaerts M, Bastings E (1994). High-dose riboflavin as a prophylactic treatment of migraine: results of an open pilot study. Cephalalgia.

[B31] Schoenen J, Jacquy J, Lenaerts M (1998). Effectiveness of high-dose riboflavin in migraine prophylaxis. A randomized controlled trial. Neurology.

[B32] Rozen TD, Oshinsky ML, Gebeline CA, Bradley KC, Young WB, Shechter AL, Silberstein SD (2002). Open label trial of coenzyme Q10 as a migraine preventive. Cephalalgia.

[B33] Majamaa K, Rusanen H, Remes AM, Pyhtinen J, Hassinen IE (1996). Increase of blood NAD+ and attenuation of lactacidemia during nicotinamide treatment of a patient with MELAS syndrome. Life Sci.

[B34] Okada H, Araga S, Takeshima T, Nakashima K (1998). Plasma lactic acid and pyruvic acid levels in migraine and tension-type headache. Headache.

[B35] Gedegbeku CA, Dhandayuthapani A, Shrayyef MZ, Egan BM (2003). Hemodynamic effects of nicotinic acid infusion in normotensive and hypertensive subjects. Am J Hypertens.

[B36] Mills E, Prousky J, Raskin G, Gagnier J, Rachlis B, Montori VM, Juurlink D (2003). The safety of over-the-counter niacin. A randomized placebo-controlled trial. BMC Clin Pharmac.

[B37] Etchason J, Miller TD, Squires RW, Allison TG, Gau GT, Marttila JK, Kottke BA (1991). Niacin-induced hepatitis: a potential side effect with low-dose time-release niacin. Mayo Clin Proc.

[B38] Rivin AU (1959). Jaundice occurring during nicotinic acid therapy for hypercholesterolemia. JAMA.

[B39] Pardue WO (1961). Severe liver dysfunction during nicotinic acid therapy. JAMA.

[B40] Einstein N, Baker A, Galper J, Wolfe H (1975). Jaundice due to nicotinic acid therapy. Am J Dig Dis.

[B41] Patterson DJ, Dew EW, Gyorkey F, Graham DY (1988). Niacin hepatitis. South Med J.

[B42] Clementz GL, Holmes AW (1987). Nicotinic acid-induced fulminant hepatic failure. J Clin Gastroenterol.

[B43] Christensen NA, Achor RWP, Berge KG, Mason HL (1961). Nicotinic acid treatment of hypercholesterolemia: comparison of plain and sustained-action preparations and report of two cases of jaundice. JAMA.

